# Metagenomic sequencing revealed the potential of banknotes as a repository of microbial genes

**DOI:** 10.1186/s12864-021-07424-5

**Published:** 2021-03-11

**Authors:** Jun Lin, Wenqian Jiang, Lin Chen, Huilian Zhang, Yang Shi, Xin Liu, Weiwen Cai

**Affiliations:** 1 Institute of Applied Genomics, Fuzhou University, No.2 Xueyuan Road, Fuzhou, 350108 China; 2 School of Basic Medical Sciences, Fujian Medical University, No.1 Xuefubei Road, Fuzhou, 350122 China; 3 College of Biological Science and Engineering, Fuzhou University, No.2 Xueyuan Road, Fuzhou, 350108 China; 4 Fujian Key Laboratory of Marine Enzyme Engineering, Fuzhou University, No.2 Xueyuan Road, Fuzhou, 350108 China

**Keywords:** Metagenomic sequencing, Banknote microbial diversity, Microbial gene variants, Superoxide dismutase gene

## Abstract

**Background:**

Genetic resources are important natural assets. Discovery of new enzyme gene sequences has been an ongoing effort in biotechnology industry. In the genomic age, genomes of microorganisms from various environments have been deciphered. Increasingly, it has become more and more difficult to find novel enzyme genes. In this work, we attempted to use the easily accessible banknotes to search for novel microbial gene sequences.

**Results:**

We used high-throughput genomic sequencing technology to comprehensively characterize the diversity of microorganisms on the US dollars and Chinese Renminbis (RMBs). In addition to finding a vast diversity of microbes, we found a significant number of novel gene sequences, including an unreported superoxide dismutase (SOD) gene, whose catalytic activity was further verified by experiments.

**Conclusions:**

We demonstrated that banknotes could be a good and convenient genetic resource for finding economically valuable biologicals.

**Supplementary Information:**

The online version contains supplementary material available at 10.1186/s12864-021-07424-5.

## Background

Paper money or banknote, as a convenient medium of payment was first issued during the Song Dynasty of China in the eleventh century. The concept of banknote was introduced to Europe in the thirteenth century and the first European banknotes were issued by a Swedish bank in 1661. Today, there are over 200 kinds of paper money in circulation in more than 200 independent countries and regions. The widespread use of mobile devices and the rise of electronic payment platforms such Applepay or Alipay, as well as bitcoin in recent years have significantly diminished the role of paper money and set a trend to phase out paper money completely in payment transactions.

Paper banknotes are prone to contamination due to frequent human contact. Of particular concern are contagious microbial contaminants that pose serious health hazard [1–3]. Paper based banknotes are excellent substrates for the attachment of microbes and for absorption of various contaminants that can provide nutrition for microbial growth. China has about 1.4 billion people [4], with huge amount of paper money (RMB) in circulation. The United States is the world’s dominant economic power [5], with its dollars circulating around the world. Thus, the RMB and the dollar’s microbiological eco-system, has a certain “representative meaning”.

Banknotes, especially US dollar, after being brought in circulation, may travel across many countries, pass thousands of different hands, experience many climatic environments before they are judged unfit for circulation and destructed. Therefore, it is meaningless to describe the microbial eco-system on each banknote or a selected set of banknotes. Our purpose of this study is to get an overview of the diversity of species on banknotes, and to explore the possibility of using paper money as economically valuable microbial genetic resources.

## Results

### NGS sequencing and data processing

**Fig. 1:**
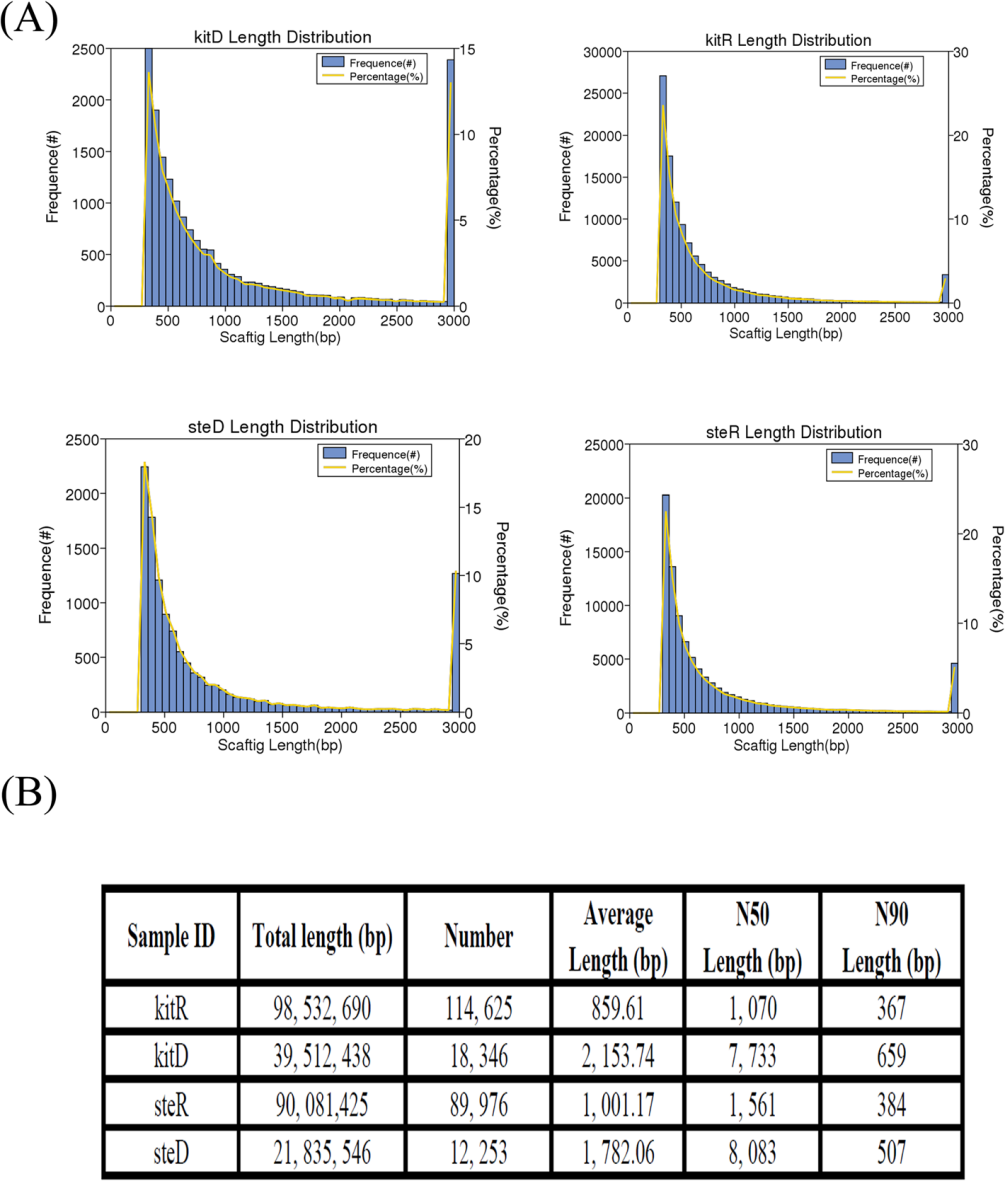
Length statistics of four scaftigs. **a**, The distribution of scaftigs length in each sample is calculated and plotted, the longitudinal axis (frequency(#)) represents the number of scaftigs and percentage (%)) represents the percentage of scaftigs number (yellow curve). The horizontal axis represents the scaftigs length. **b**, SampleID indicates the name of the sample; Total Length (bp), the overall length of the assembled scaftigs; Number, the total number of scaftigs assembled; Average Length (bp), the average length of the assembled Scaftigs; N50 and N90 statistic defines assembly quality in terms of contiguity [6]

We used Next Generation Sequencing (NGS) to obtain sequencing reads from metagenomic DNA isolated from banknotes. The sequencing mode was PE 125:125. Sample SteR, KitD, KitR, and SteD produced 4.93 Gb, 4.94 Gb, 4.94 Gb and 5.45 Gb raw bases, respectively. Clean bases of SteR, KitD, KitR, and SteD were 4.89 Gb, 4.78 Gb, 4.79 Gb and 5.41 Gb raw bases, respectively. The Clean_Q30 values, defined as the percentage of bases in clean data with sequencing error rate less than 0.001, were 92.19, 90.91, 93.51 and 91.16% for sample SteR, KitD, KitR, and SteD, respectively. All raw data were uploaded to the NCBI-SRA database under the accession number of SRP128023. All scaftigs in the assembled results were counted as well as the distribution of scaftigs’ length in each sample. The statistical results are shown in Fig. 1.

We analyzed the alpha diversity index (shannon, simpson, chao1, goods_coverage) of different samples at a 97% consistency threshold (Table 1). The results (Table 1) showed that for either KitWe analyzed the alpha diversity index (shannon, simpson, chao1, goods_coverage) of different samples at a 97% consistency threshold (Table 1). The results (Table 1) showed that for either Kit or STE extraction method, Chao1 value, Shannon and simpson indexes of the RMB samples were significantly greater than the respective index of the dollar samples. It is noticeable that for either kit or STE extraction method, the N50 and N90 of dollar samples were significantly greater than the respective N50 and N90 value of the RMB samples. This is mainly due to the presence of more microbial species on the RMBs.

**Table 1:** Alpha indexes statistics

	observed_species	Shannon^**1**^	Simpson^**2**^	Chao1^**3**^	goods_coverage^**4**^
kitR	891	4.385124	0.846254	891	1
kitD	315	3.077936	0.689636	315	1
steR	886	4.706317	0.874302	886	1
steD	289	1.908215	0.54075	289	1

^1^ The richness and evenness of the community were considered. The higher the Shannon index, the higher the community diversity

^2^ The probability that two randomly sampled individuals belong to different species = 1-the probability that two randomly sampled individuals belong to the same species. The greater the Simpson index, the higher the community diversity

^3^ Chao1 algorithm is used to estimate the number of OTUs in the community. The larger the Chao1 value, the more the total number of species

^4^ Sequencing depth index

### Microbial diversity on banknotes

**Fig. 2:**
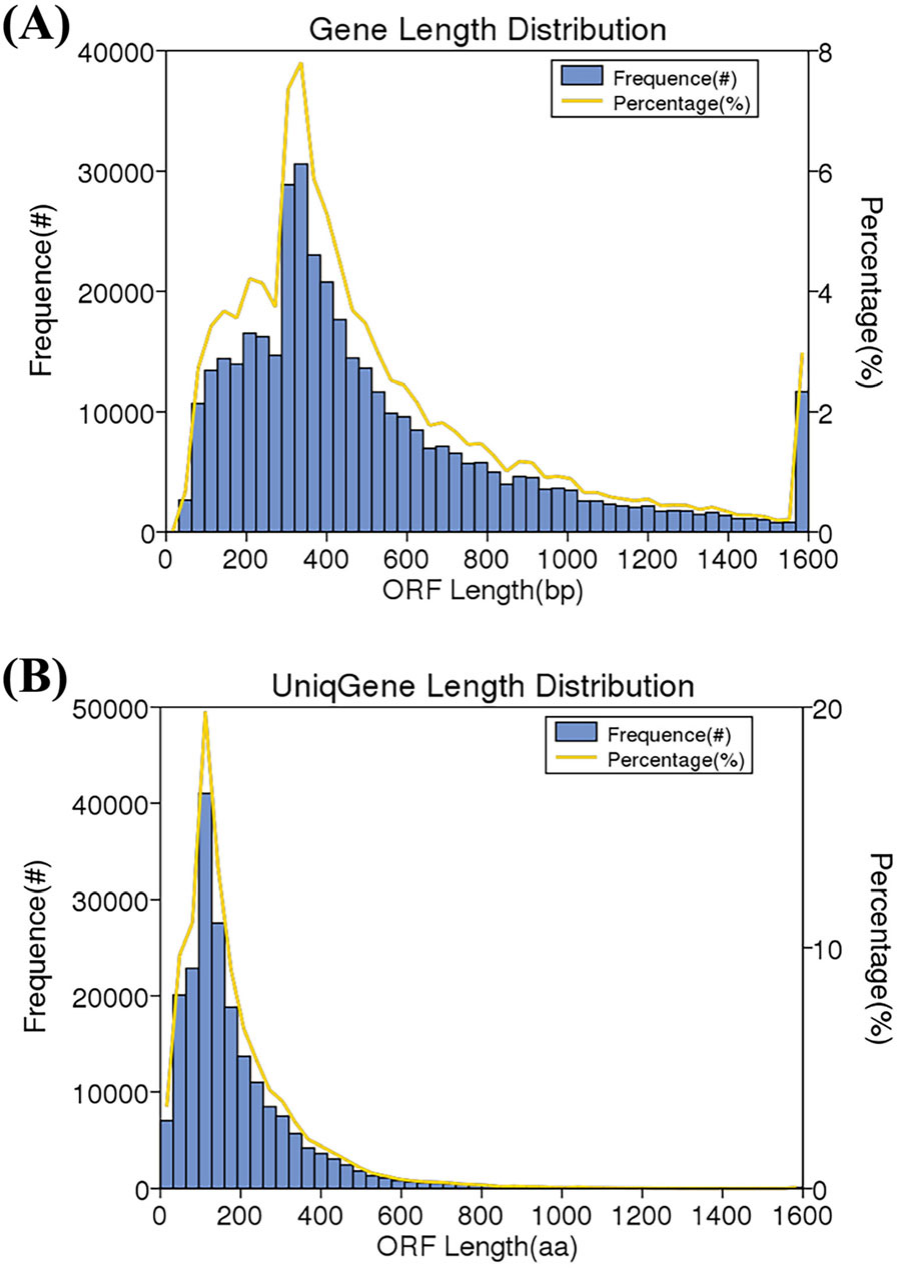
The sequence length distribution for de novo detected gene’s ORFs and non-redundant unigene’s ORFs. A, The length distribution of predicted gene sequences for all 4 samples. The longitudinal axis shows the number of predicted genes (in blue) and the percentage of the predicted gene number (yellow curve). The horizontal axis represents length of the predicted genes (in bp). B, Non-redundant protein sequence length distribution statistics for all 4 samples. The longitudinal axis frequency (#), the number of genes and percentage (%), the percentage of the number of genes (yellow). The horizontal axis, the protein amino acid sequence length of the ORF.

From a total of 20 Gb raw sequence data, we identified 392,211 ORFs. After removing redundant sequences, we identified a total of 207,051 unigene sequences. The sequence length statistics is shown in Fig. 2. Majority of the predicted gene sequences are between 300 and 400 bp, among which most of predicted gene sequences range from 330 bp to 360 bp (Fig. 2a). The length of most non-redundant protein sequences is between 35 and 210 amino acids, among which the length of most non-redundant protein sequences is in the range 100–130 amino acids, accounting for about 16% (Fig. 2b).

### Gene function annotation

**Fig. 3:**
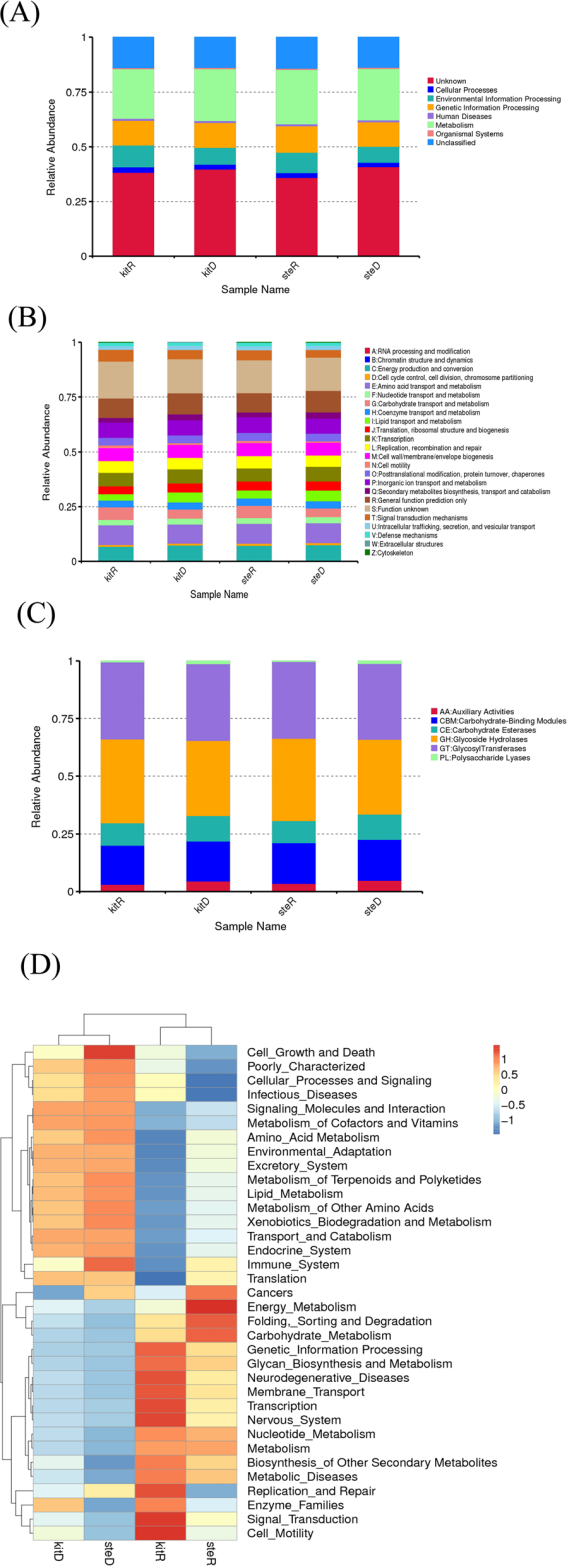
Pathway annotation based on KEGG, CAZy, eggNOG databases and abundance heatmap of KEGG annotated gene functions. A, B, C, the results of KEGG, eggNOG and CAZy annotation respectively, the functions of genes of each sample are graphically tabulated. The horizontal axis represents different samples, and the vertical coordinate, the relative abundance of the genes of a certain function. D, Functional annotation and abundance information of all samples based on KEGG, we selected the first 35 of the functions ranked by abundance in each sample to construct a hot map (Kegg Select the second level (Levels 2), from the functional information and the difference between the sample by two levels of clustering

We performed gene function annotation for the identified 207,051 unigenes using the CAZy [7], eggNOG [8] and KEGG [9] database, and statistical results are summarized as shown in Fig. 3.

We found that using the KEGG database, 25% of the pathway genes are related to metabolism, 11% related to genetic information processing, 9% of annotated genes are involved in environmental information processing, and about 50% of genes are of unknown and unclassified functions (Fig. 3a, Table 2). When the eggNOG database was used for function annotation we found a variety of metabolism related pathway genes, including Inorganic ion transport and metabolism, amino acid transport and metabolism, nucleotide transport and metabolism, carbohydrate transport and metabolism, coenzyme transport and metabolism and lipid transport and metabolism (Fig. 3b, Table 3). Using the CAZy database for function annotation, we found a large number of glycosyl transferases and glycoside hydrolases (Fig. 3c, Table 4).

**Table 2:** Proportion of gene functions annotated using the KEGG database for the samples

Description	kitR	kitD	steR	steD
Genetic information processing	11.28%	11.36%	12.10%	11.19%
Unclassified	14.05%	13.93%	14.32%	13.85%
Metabolism	22.50%	23.56%	24.70%	23.34%
Environmental information processing	9.99%	7.71%	9.30%	7.29%
Unknown	38.25%	39.76%	35.90%	40.83%
Others	3.93%	3.68%	3.68%	3.50%

**Table 3:** Proportion of gene functions annotated using the eggNOG database for the 4 samples

Description	kitR	kitD	steR	steD
P: Inorganic ion transport and metabolism	6.94%	6.88%	7.17%	6.88%
E: Amino acid transport and metabolism	9.01%	8.72%	9.07%	9.09%
I: Lipid transport and metabolism	2.88%	4.57%	3.68%	4.92%
F: Nucleotide transport and metabolism	2.46%	2.69%	2.61%	2.82%
G: Carbohydrate transport and metabolism	5.76%	4.15%	5.64%	3.92%
H: Coenzyme transport and metabolism	3.10%	3.25%	3.34%	3.30%
S: Function unknown	16.84%	15.59%	15.03%	15.22%
Others	53.01%	54.15%	53.46%	53.85%

**Table 4:** Proportion of gene functions for the 4 samples using the CAZy database

Description	kitR	kitD	steR	steD
GH: Glycoside Hydrolases	36.27%	32.47%	35.61%	32.34%
GT: Glycosyl Transferases	33.48%	33.36%	33.36%	32.92%
Others	30.25%	34.17%	31.03%	34.74%

### Banknotes as a genetic resource

Banknotes in circulation are exposed to a variety of environments and are expected to carry a diversity of microbes. Some of these microbes may be a good genetic resource of potential economic value. To explore such possibility, we further analyzed the 207,051 non-redundant unigene data for enzyme coding sequences. The 207,051 non-redundant unigene dataset was annotated with KEGG with an E-value threshold of 10^− 5^. Among the 350 enzymes in the Enzyme Commission EC number at Sub-subclasses level, we found a total of 225 enzyme sequences in the banknote metagenomic data. Some of these enzyme genes are of high economic value, such as SOD, which is an enzyme widely used in cosmetics and medicine, amylase, endoglucanase and beta-D-glucodidase, penicillin amidase, polyketide synthase, and nonribosomal peptide synthetases (NRPSs), which are large multi-modular biocatalysts that utilize complex regiospecific and stereospecific reactions to assemble structurally and functionally diverse peptides of important medicinal applications [10]. Several of these enzymes are common enzymes of industrial and medical value (Table 5).

**Table 5:** Overview of seven important enzymes found in this study

Name	Ec number	The number of Gene in total	KEGG Identity < 50%	50% ≤ KEGG Identity ≤ 90%	KEGG Identity > 90%	Function
SOD	1.15.1.1	61	0	33	28	Catalyzes the dismutation (or partitioning) of the superoxide (O_2_−) radical into either ordinary molecular oxygen (O_2_) or hydrogen peroxide (H_2_O_2_). It is useful in the food and cosmetic industry.
Alpha-amylase	3.2.1.1	40	0	30	10	Hydrolyses alpha bonds of large, alpha-linked polysaccharides, useful in the food industry.
Penicillin amidase	3.5.1.11	48	3	26	19	Used in the production of beta lactam antibiotic intermediates.
Polyketide synthase	2.3.1.-	1699	78	929	692	A family of multi-domain enzymes or enzyme complexes that produce polyketides, a large class of secondary metabolites
Non-ribosomal peptide synthetase	6.3.2.-	488	17	265	206	Nonribosomal peptides (NRP) are a class of peptide secondary metabolites. Nonribosomal peptides are a very diverse family of natural products with an extremely broad range of biological activities and pharmacological properties.
Endoglucanase	3.2.1.4	57	9	45	3	Catalyzes cellulolysis
Beta-D-Glucodidase	3.2.1.21	136	4	99	33	Catalyzes the hydrolysis of the glycosidic bonds

We also found a large number of suspected but unreported novel enzyme genes on the banknotes. These enzymes may have activities and functions that can be explored for new applications.

Since sequences were acquired by de novo sequencing and assembled by software, many of the identified enzyme genes may not be real existence. To evaluate these data as a genetic resource for novel enzymes, we chose one from these identified enzymes for protein expression.

### Expression of a novel SOD enzyme

Superoxide dismutase or SOD is an important oxygen free radical scavenger, existing in most living cells exposed to oxygen [11]. It is an important pharmaceutical enzyme and cosmetic additive. Due to its high economic value and important role in disease processes, this enzyme has been extensively studied since its discovery in 1969 [11] and numerous natural SOD enzyme gene variants have been reported [12].

**Fig. 4:**
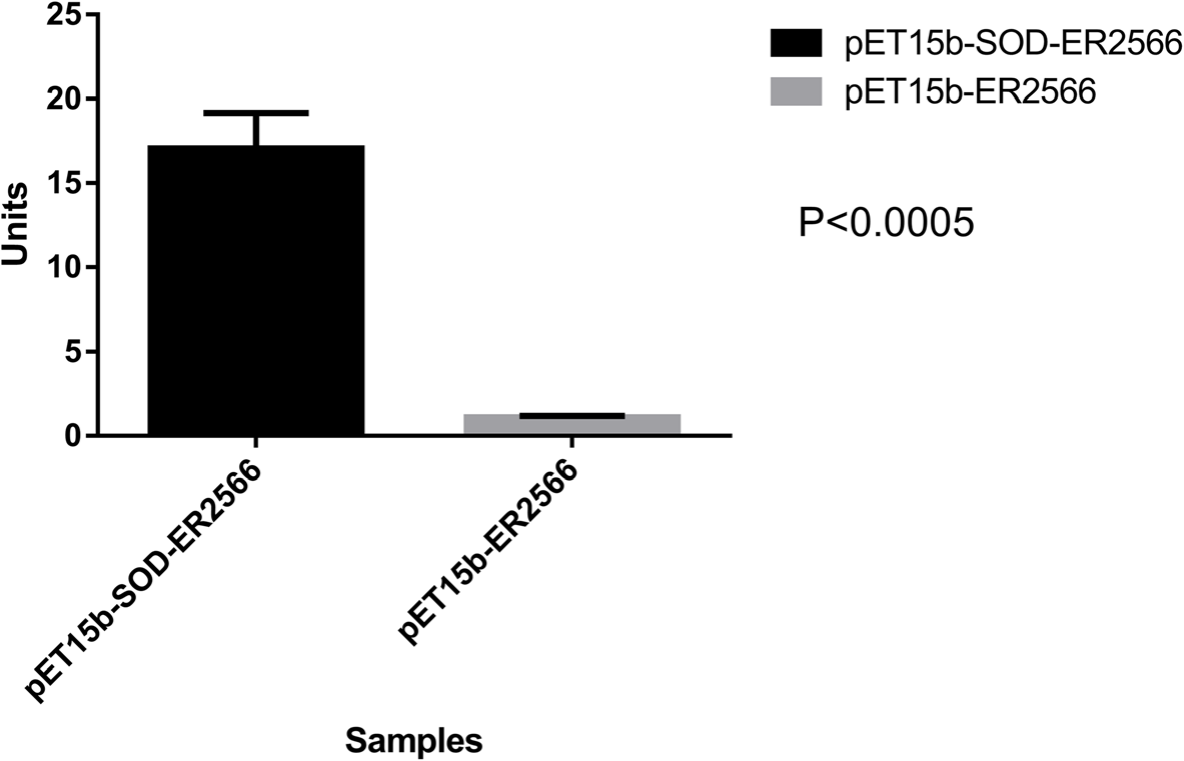
The expressed activity of a SOD enzyme candidate. The vertical axis is the enzyme activity, the horizontal axis represent the SOD enzyme candidate expressing cassette ET15b-SOD-ER2566 (A) and the blank cloning vector PET15b-ER2566 (B)

**Fig. 5:**
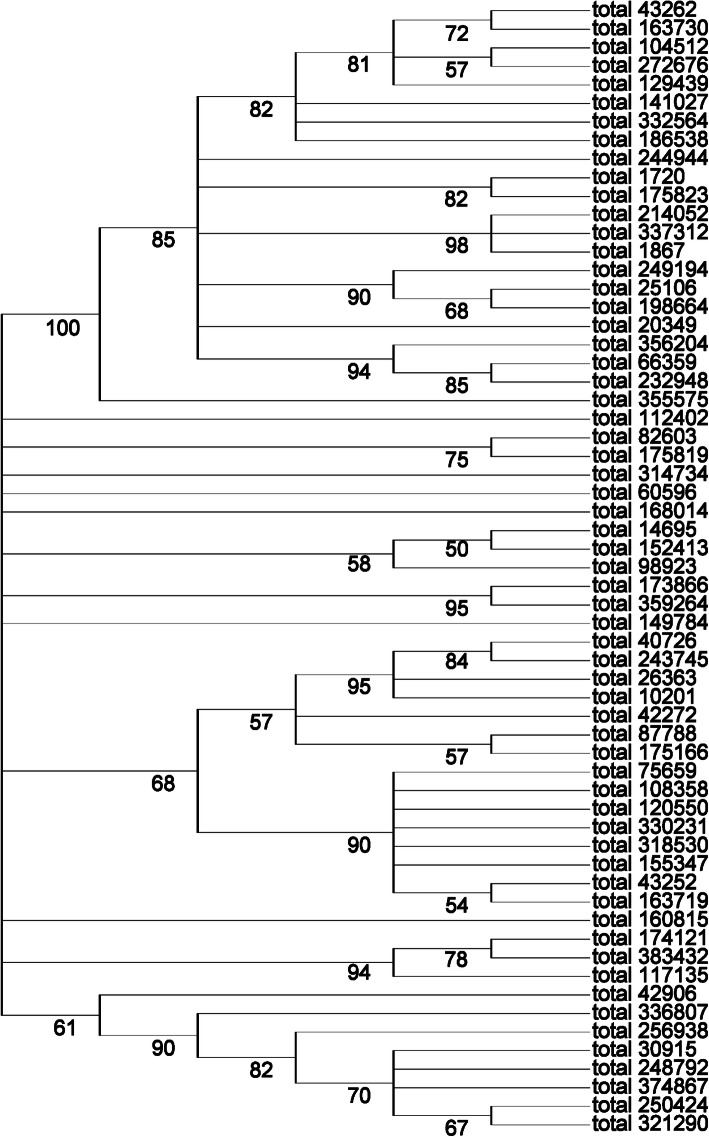
Molecular Phylogenetic analysis of SOD genes. The total_314734 and other SOD proteins present in our dataset were constructed by maximum likelihood method [14]. The numbers above the branch points denote the confidence levels of the relationship of the clustered sequences determined by boot strap statistical analysis [15]

In the KEGG annotation of sequences, we found a sequence, numbered total_314734, with only 60% nucleotide identity and 76% protein sequence similarity to the SOD genes using the NCBI online protein Blast program (Database version: March 2017). We suspected this is an unreported SOD enzyme gene sequence. We obtained the full length sequence of this gene by direct PCR using the paper money’s metagenomic DNA as template. All primers used in this article was shown in the Supplemental Table S1. We used the *E. coli* pET expression system [13] to obtain the recombinant protein. It turned out that the expressed protein had a strong SOD activity using a SOD activity assay kit (Beyotime, China) (Fig. 4). In this specific case, we demonstrated that the metagenome of banknotes could be a potentially important genetic resource for finding novel genes of great economic value. In addition, we performed phylogenetic analysis of amino acid sequences of this enzyme. The result was shown in Fig. 5. The sequence of total_314734 was submitted to Genbank under the accession number of MK681865.

All obtained SOD sequences in our data were translated to amino acid sequences and analysis with MEGA7 [16] to construct the phylogenetic tree (Fig. 5). The novel SOD sequence of total_314734 was classified into a unique branch, with a low homology to others. We could draw the conclusion that there is a rich diversity of SOD gene variants on banknotes and these SOD genes came from different family and may have valuable properties and applications.

## Discussion

The number of non-redundant genes per GB base of raw sequence we found on banknotes was more than that of the intestinal [17] and soil [18]. Of note is that the amount of raw sequence data in this study is much lower than that of previous studies and the number of samples was far less (Table 6). This may indicate that our findings could be only a very small fraction of the whole microbiota on banknotes.

**Table 6:** Comparison of Gene abundance of fecal samples, soil, sea water and this study

sample	raw data	mapped genes number	Gene density	reference
Fecal samples of 124 European adults	576.7Gb	mapped to the 319,812 target genes	555 target genes/GB	QIN J, LI R, RAES J, et al. A human gut microbial gene catalogue established by metagenomic sequencing [J]. Nature, 2010, 464(7285): 59–65.
Two large soil studies	398 Gb	3,533 unique KO identifiers	9 unique KO identifiers/GB	HOWE A C, JANSSON J K, MALFATTI S A, et al. Tackling soil diversity with the assembly of large, complex metagenomes [J]. Proc Natl Acad Sci U S A, 2014, 111(13): 4904–4909.
12 RMB and 12 dollar	20 Gb	392,211 ORFs, 207,051 unigene	19,611 ORFs/GB, 10,353 unigene/GB	this study

From metabolism analysis of the KEGG annotation (Fig. 3a), we found that cell motility, signal transduction, membrane transport related pathway were very active. This suggests that the microbes on the banknotes might form a certain social network to adapt to the special environment on banknotes. Metabolic pathways of DNA replication and repair, energy metabolism, carbohydrate metabolism, and amino acid metabolism were also very active as expected. These activities are essential to maintain the survival and reproduction of microbial cells. We also found common pathway genes related to cell survival, amino acid metabolism, energy metabolism, as well as cell structure maintenance. These findings suggest that there is a whole eco-system on banknotes to support microbial life activities and biodegradation.

It is no surprise that banknotes contain a rich diversity of microbes. However, the abundance of enzyme genes found in this study was still unexpected, considering that the data were derived from only 24 banknotes. There are precedents of identifying nonel enzyme genes from a metagenomic library [19]. For example, economically valuable enzymes such as lipase and esterase have been isolated from soil and sea water samples [20]. Charlop-Powers [21] found that Urban Park soil microbiomes are a rich reservoir of natural product biosynthetic diversity in New York’s park soils. Many of the putative enzyme sequences have a low identity value with previously identified sequences in the public databases, as exemplified by our discovery of a novel SOD enzyme gene variant, which was successfully expressed and shown to have activity. These enzymes may have unusual activity and tolerance and potentially can be harnessed for some special purposes and occasions. We also found thousands of non-ribosomal peptide synthetases and polyketide synthases, and many are suspected novel variants of these two enzymes. These two enzymes are the key enzymes for the production of various economically valuable compounds.

## Conclusions

This work showed that banknotes are a good and convenient genetic repository of high economic value. At present, the genetic resources of terrestrial microbes are thought to have been extensively explored. The ocean is considered the last treasure trove of new life and new genetic resources. Our findings indicated that globally circulating banknotes may be a new territory which can be explored for new genetic resources.

## Methods

### Sample preparation

We collected RMB in China and US dollars in the United States, one in the eastern hemisphere and the other in the western hemisphere. The dollar samples and the RMB samples are treated separately, to avoid cross contamination. In this study, we collected 12 one Yuan bills of RMB in China, and 12 one dollar bills in the United States. The surface of each bill was washed with sterile water, and the liquid was filtered through a 0.22 μm filter to collect the microbes. Extraction of metagenome was performed for high throughput sequencing. In order to obtain the most complete information on the metagenomic DNA, We used two genomic DNA extraction methods (Supplemental Methods S1), the classic STE buffer (sodium chloride, Tris-HCl, EDTA) and Mobio kit, to isolate bacterial genomic DNA from banknotes. The STE is suitable for bacteria, especially Gram negative strains. The kit from Mobio is advantageous for some tough-to-lyse microbes. But the harsh cell grinding and disrupting procedure in this method may damage the genomic DNA of some fragile microbes. In this study, four DNA samples of the metagenome were studied, which were labeled as follow: steD: metagenomic DNA from dollars extracted using STE method; KitD: metagenomic DNA from dollars using Mobio Kit; SteR; metagenomic DNA from RMB using STE method; KitR: metagenomic DNA from RMB using Mobio Kit. The extracted DNA samples were sequenced and analyzed separately.

### Sequencing

A total amount of 1 μg metagenomic DNA per sample was used as input material for preparation of DNA libraries. Sequencing libraries were generated using NEBNext® Ultra™ DNA Library Prep Kit for an Illumina Hiseq2500 sequencer (NEB, USA) following manufacturer’s recommendations and index codes were added to mark sequences for each sample. Briefly, the DNA sample was fragmented by sonication to an average size of 300 bp, then DNA fragments were end-polished, A-tailed, and ligated with the full-length adaptor. PCR amplification was performed on the ligated products using an adaptor specific primer pair. PCR products were purified (AMPure XP system) and libraries were analyzed for size distribution by Agilent 2100 Bioanalyzer and quantified using real-time PCR. An Illumina Hiseq2500 sequencer was used for high-throughput sequencing of the four DNA samples and paired-end reads were generated. The bioinformatics analysis method for NGS data of this study was shown in the Supplemental Methods S2.

### Alpha diversity analysis

The Alpha diversity index analysis is based on the results of assembly for species annotation analysis, for which the scaftigs data was used. The command (alpha_diversity.py -i /TJPROJ1/MICRO/NGS_project_2020/yaoyuanyuan/X101SC19090394-Z01/X101SC19090394-Z01-J013/report_20200527/report2/03.Make_OTU/otu97/Table_Stats/sorted_otu_table.biom -m observed_species,shannon,simpson,chao1,ACE,goods_coverage,PD_whole_tree -t /TJPROJ1/MICRO/NGS_project_2020/yaoyuanyuan/X101SC19090394-Z01/X101SC19090394-Z01-J013/report_20200527/report2/03.Make_OTU/otu97/OTU_Trees/rep_set.tre -o alpha_diversity.txt 2 > res.log) of Qiime software (version 1.9.1) was used to calculate observed OTUs, Chao1, Shannon, Simpson, goods coverage index.

### Molecular cloning and expression of SOD enzyme

pET15b plasmid (Novagen, USA) was digest with BamHI restriction enzyme (Thermo, USA) first to get the linear DNA product. The target gene was amplification using PCR with 2 × Taq Plus MasterMix (CWBIO, China). Both the linearized plasmid and PCR product were loaded to an agarose gel for separation, Gel bands of correct size were cut out. A gel DNA recovery step was performed with a GeneJet Extraction and DNA Cleanup Micro Kit (Thermo, USA). The linearized pET15b and SOD gene was mix and treated by an In-Fusion HD Cloning Kit (Takara, Japan) to ligate them. The recombinant plasmid was confirmed by Sanger sequencing and then transformed into *E. coli* ER2566 by heat shock method. This engineered strain was cultured in LB medium and induced to express the target gene by IPTG induction. Subsequently, all bacteria cells was collected and disrupted by sonication. The SOD activity was tested using a commercial kit (Beyotime, China).

### Molecular phylogenetic analysis

The evolutionary history was inferred by using the Maximum Likelihood method based on the Poisson correction model (Zuckerkandl and Pauling 1965). The bootstrap consensus tree inferred from 70 replicates is taken to represent the evolutionary history of the taxa analyzed. Branches corresponding to partitions reproduced in less than 50% bootstrap replicates are collapsed. The percentage of replicate trees in which the associated taxa clustered together in the bootstrap test (70 replicates) are shown next to the branches (Felsenstein 1985). Initial tree(s) for the heuristic search were obtained automatically by applying Neighbor-Join and BioNJ algorithms to a matrix of pairwise distances estimated using a JTT model, and then selecting the topology with superior log likelihood value. The analysis involved 61 amino acid sequences. Evolutionary analyses were conducted in MEGA7 (Kumar et al. 2016b).

## Supplementary Information

**Additional file 1: Supplemental Methods S1.** STE DNA extraction method. Steps of extracting metagenomic DNA by STE extraction method.

**Additional file 2: Supplemental Methods S2.** Informatic analysis of metagenomes of banknotes. The bioinformatics analysis method for NGS data of this study.

**Additional file 3: Supplemental Table S1.** Primer sequence. Primer sequences used in this paper.

## Data Availability

All raw data was uploaded to the NCBI-SRA database under the accession number of SRP128023. The sequence of total_314734 was submitted to Genbank under the accession number of MK681865.
