# Supplementary material for: Metagenomic sequencing revealed the potential of banknotes as a repository of microbial genes
Source: BMC Genomics. 2021 Mar 11;22:173. doi: 10.1186/s12864-021-07424-5 (PMC7953773; doi:10.1186/s12864-021-07424-5)
Supplement: Supplementary file 1 — Additional file 1: Supplemental Methods S1. STE DNA extraction method. Steps of extracting metagenomic DNA by STE extraction method. [file 12864_2021_7424_MOESM1_ESM.doc]

**STE DNA extraction method**

1. Banknotes were rinsed with distilled water, which was collected and passed through a filter membrane. The filter membrane was ground up in a 2 ml tube with a micro pestle in 600 µl 1X STE buffer (50 mM NaCl, 50 mM Tris-HCL, 100 mM EDTA, pH 8.0) along with 60 µl lysozyme (200 mg/ml). The mixture was incubated for 30 minutes at 37 ℃.

2. Add 1% SDS, 6 µl RNase A (20 mg/ml), 12 µl Proteinase K (20 mg/ml) and mix it.

3. Lyse for 30 minutes at 65 ℃. Gently mix the tube by inverting every 5 minutes.

4. Centrifuge at 12,000g for 10 minutes at 4 ℃. Transfer the supernatant to a fresh tube.

5. Add equal volume of Tris saturated phenol mix and centrifuge at 12,000g for 10 minutes at 4 ℃. Transfer the supernatant to a fresh tube.

6. Then add equal volume of chloroform: isoamylalcohol (24:1) and centrifuge at 12,000g for 10 minutes at 4 ℃.

7. Transfer the clear supernatant to a fresh tube, add two fold volume cold ethanol and keep it for 1 hour at -20 ℃.

8. Centrifuge at 12,000g for 10 minutes at 4 ℃ and then remove the supernatant.

9. Wash the pellet with 70% ethanol.

10. Keep the pellet at 37 ℃ for 10 minutes.

11. Dissolve the dry pellet in 100 µl nuclease free water. Store the isolated DNA at -20℃.
