# Supplementary material for: Metagenomic sequencing revealed the potential of banknotes as a repository of microbial genes
Source: BMC Genomics. 2021 Mar 11;22:173. doi: 10.1186/s12864-021-07424-5 (PMC7953773; doi:10.1186/s12864-021-07424-5)
Supplement: Supplementary file 2 — Additional file 2: Supplemental Methods S2. Informatic analysis of metagenomes of banknotes. The bioinformatics analysis method for NGS data of this study. [file 12864_2021_7424_MOESM2_ESM.docx]

**Informatic analysis of metagenomes of banknotes**

Metagenomic analysis can more truly reflect the microbial composition and interaction in the sample, and study the metabolic pathway and gene function at the molecular level. (Handelsman *et al*. 1998; Chen and Pachter 2005; Tringe and Rubin 2005; Tringe *et al*. 2005).

1. Preprocessing of sequencing data

The Raw Data obtained from the Illumina HiSeq sequencing platform was preprocessed using Readfq (https://github.com/lh3/readfq) to obtain the Clean Data for subsequent analysis.

2. Metagenome Assembly

The Clean Data was assembled and analyzed using the SOAPdenovo software (Luo *et al*. 2012) with parameters (Scher *et al*. 2013; Qin *et al*. 2014; Brum *et al*. 2015; Feng *et al*. 2015) set as follows: -d 1, -M 3, -R, -u, -F, -K 55; then split the assembled Scaftigs at N and to generate Scaftigs without N (Mende *et al*. 2012; Nielsen *et al*. 2014; Qin *et al*. 2014).

3. Taxonomy prediction

1) The [USEARCH](http://www.drive5.com/usearch/manual/dereplication.html) software was used to reduce the redundancy of Scaftigs from each sample (Edgar 2010). The Scaftigs were blasted against the sequences of Bacteria, Fungi, Archaea and Viruses, which were all extracted from the NT database (Version: 2014-10-19, [https: //www.ncbi.nlm.nih.gov/) of](https://www.ncbi.nlm.nih.gov/)) NCBI with the parameter setting as blastp, -e 1e-5.

2) For the finally aligned results of each sequence, as each sequence may have multiple aligned results, we used the LCA algorithm, the software used in MEGAN (Huson *et al.* 2011) for system classification, to annotate the sequences for the species assignment.

3) The table containing the number of genes and the abundance information of each sample in each taxonomy hierarchy (kingdom, phylum, class, order, family, genus, species) were obtained based on the LCA annotation results and the gene abundance table.

4. Gene prediction and abundance analysis

1) The ORF of assembled Scaftigs (≥300 bp) (Yok and Rosen 2011) were fed into MetaGeneMark (Zhu *et al*. 2010) software for gene prediction.

2) For the predicted ORFs, CD-HIT (Li and Godzik 2006; Fu *et al*. 2012) software was used to remove redundant predictions and obtain the unique initial gene catalogue (Sunagawa *et al*. 2015), the parameters option (Zeller *et al*. 2014; Sunagawa *et al*. 2015) are -c 0.95, -G 0, -aS 0.9, -g 1, -d 0.

3) Based on the number of mapped reads and the gene length, the abundance information of each gene in each sample was calculated using the formula as follows:

$$G_{k}=\frac{r_{k}}{L_{k}}\cdot\frac{1}{\sum_{i=1}^{n} \frac{r_{i}}{L_{\begin{matrix} i \\ \end{matrix}}}}$$

where r represents the number of reads mapped to the genes and L represents gene’s length (Qin *et al*. 2010; Karlsson *et al*. 2012; Cotillard *et al*. 2013; Le Chatelier *et al*. 2013; Zeller *et* *al*. 2014; Villar *et al*. 2015).

4) The basic information statistics, core-pan gene analysis, correlation analysis of samples and Venn figure analysis of number of genes are all based on the abundance of each gene in each sample in gene catalogue.

5. Common functional database annotations

1) Unigenes blasted against a functional database with the parameter setting of blastp, -e 1e-5 (Li *et al*. 2014; Feng *et al*. 2015). Functional databases include KEGG (Kanehisa *et al*. 2006; Kanehisa *et al*. 2014) database (Version 58, <http://www.kegg.jp/kegg/>), eggNOG (Powell *et al*. 2014) database (Version 4.0, [http://eggnogdb.embl.de/#/app/home](http://eggnogdb.embl.de/%23/app/home)), CAZy (Cantarel *et al*. 2009) database (Version 20141125, <http://www.cazy.org/>). For each sequence’s blast result, the best Blast Hit is used for subsequent analysis (Li *et al*. 2014; Backhed *et al*. 2015; Feng *et* *al.* 2015).

2) Statistics of the relative abundance of different functional hierarchy. The relative abundance of each functional hierarchy equals the sum of relative abundance annotated to that functional level.

3) Based on the function annotation result and gene abundance table, the gene number table of each sample in each taxonomy hierarchy is obtained.

Readfq (V5, https://github.com/cjfields/readfq)

SOAP denovo (Version: 2.21): <http://soap.genomics.org.cn/soapdenovo.html>

USEARCH (Version: 7.0.1001): <http://www.drive5.com/usearch/manual/dereplication.html>

SoapAligner (Version: 2.21): <http://soap.genomics.org.cn/soapaligner.html>

MetaGeneMark (Version: 2.10): <http://exon.gatech.edu/GeneMark/metagenome/Prediction>

CD-HIT (Version: 4.5.8): <http://www.bioinformatics.org/cd-hit/>

References

Backhed F, Roswall J, Peng Y, Feng Q, Jia H, Kovatcheva-Datchary P, Li Y, Xia Y, Xie H, Zhong H et al. 2015. Dynamics and Stabilization of the Human Gut Microbiome during the First Year of Life. *Cell Host Microbe* **17**(5): 690-703.

Brum JR, Ignacio-Espinoza JC, Roux S, Doulcier G, Acinas SG, Alberti A, Chaffron S, Cruaud C, de Vargas C, Gasol JM et al. 2015. Ocean plankton. Patterns and ecological drivers of ocean viral communities. *Science* **348**(6237): 1261498.

Cantarel BL, Coutinho PM, Rancurel C, Bernard T, Lombard V, Henrissat B. 2009. The Carbohydrate-Active EnZymes database (CAZy): an expert resource for Glycogenomics. *Nucleic acids research* **37**(Database issue): D233-238.

Chen K, Pachter L. 2005. Bioinformatics for whole-genome shotgun sequencing of microbial communities. *PLoS Comput Biol* **1**(2): 106-112.

Cotillard A, Kennedy SP, Kong LC, Prifti E, Pons N, Le Chatelier E, Almeida M, Quinquis B, Levenez F, Galleron N et al. 2013. Dietary intervention impact on gut microbial gene richness. *Nature* **500**(7464): 585-588.

Edgar RC. 2010. Search and clustering orders of magnitude faster than BLAST. *Bioinformatics* **26**(19): 2460-2461.

Fang H, Wang HF, Cai L, Yu YL. 2015. Prevalence of Antibiotic Resistance Genes and Bacterial Pathogens in Long-Term Manured Greenhouse Soils As Revealed by Metagenomic Survey. *Environ Sci Technol* **49**(2): 1095-1104.

Feng Q, Liang S, Jia H, Stadlmayr A, Tang L, Lan Z, Zhang D, Xia H, Xu X, Jie Z et al. 2015. Gut microbiome development along the colorectal adenoma-carcinoma sequence. *Nat Commun* **6**: 6528.

Forsberg KJ, Patel S, Gibson MK, Lauber CL, Knight R, Fierer N, Dantas G. 2014. Bacterial phylogeny structures soil resistomes across habitats. *Nature* **509**(7502): 612-616.

Fu L, Niu B, Zhu Z, Wu S, Li W. 2012. CD-HIT: accelerated for clustering the next-generation sequencing data. *Bioinformatics* **28**(23): 3150-3152.

Handelsman J, Rondon MR, Brady SF, Clardy J, Goodman RM. 1998. Molecular biological access to the chemistry of unknown soil microbes: a new frontier for natural products. *Chem Biol* **5**(10): R245-249.

Huson DH, Mitra S, Ruscheweyh HJ, Weber N, Schuster SC. 2011. Integrative analysis of environmental sequences using MEGAN4. *Genome Res* **21**(9): 1552-1560.

Jia B, Raphenya AR, Alcock B, Waglechner N, Guo P, Tsang KK, Lago BA, Dave BM, Pereira S, Sharma AN et al. 2017. CARD 2017: expansion and model-centric curation of the comprehensive antibiotic resistance database. *Nucleic Acids Res* **45**(D1): D566-D573.

Kanehisa M, Goto S, Hattori M, Aoki-Kinoshita KF, Itoh M, Kawashima S, Katayama T, Araki M, Hirakawa M. 2006. From genomics to chemical genomics: new developments in KEGG. *Nucleic acids research* **34**(Database issue): D354-357.

Kanehisa M, Goto S, Sato Y, Kawashima M, Furumichi M, Tanabe M. 2014. Data, information, knowledge and principle: back to metabolism in KEGG. *Nucleic acids research* **42**(Database issue): D199-205.

Karlsson FH, Fak F, Nookaew I, Tremaroli V, Fagerberg B, Petranovic D, Backhed F, Nielsen J. 2012. Symptomatic atherosclerosis is associated with an altered gut metagenome. *Nature Communications* **3**.

Le Chatelier E, Nielsen T, Qin J, Prifti E, Hildebrand F, Falony G, Almeida M, Arumugam M, Batto JM, Kennedy S et al. 2013. Richness of human gut microbiome correlates with metabolic markers. *Nature* **500**(7464): 541-546.

Li J, Jia H, Cai X, Zhong H, Feng Q, Sunagawa S, Arumugam M, Kultima JR, Prifti E, Nielsen T et al. 2014. An integrated catalog of reference genes in the human gut microbiome. *Nat Biotechnol* **32**(8): 834-841.

Li W, Godzik A. 2006. Cd-hit: a fast program for clustering and comparing large sets of protein or nucleotide sequences. *Bioinformatics* **22**(13): 1658-1659.

Liu B, Pop M. 2009. ARDB-Antibiotic Resistance Genes Database. *Nucleic acids research* **37**: D443-D447.

Luo R, Liu B, Xie Y, Li Z, Huang W, Yuan J, He G, Chen Y, Pan Q, Liu Y et al. 2012. SOAPdenovo2: an empirically improved memory-efficient short-read de novo assembler. *Gigascience* **1**(1): 18.

Martinez JL, Coque TM, Baquero F. 2015. What is a resistance gene? Ranking risk in resistomes. *Nature reviews Microbiology* **13**(2): 116-123.

McArthur AG, Waglechner N, Nizam F, Yan A, Azad MA, Baylay AJ, Bhullar K, Canova MJ, De Pascale G, Ejim L et al. 2013. The comprehensive antibiotic resistance database. *Antimicrobial agents and chemotherapy* **57**(7): 3348-3357.

McArthur AG, Wright GD. 2015. Bioinformatics of antimicrobial resistance in the age of molecular epidemiology. *Curr Opin Microbiol* **27**: 45-50.

Mende DR, Waller AS, Sunagawa S, Jarvelin AI, Chan MM, Arumugam M, Raes J, Bork P. 2012. Assessment of metagenomic assembly using simulated next generation sequencing data. *PloS one* **7**(2): e31386.

Nielsen HB, Almeida M, Juncker AS, Rasmussen S, Li JH, Sunagawa S, Plichta DR, Gautier L, Pedersen AG, Le Chatelier E et al. 2014. Identification and assembly of genomes and genetic elements in complex metagenomic samples without using reference genomes. *Nature Biotechnology* **32**(8): 822-828.

Powell S, Forslund K, Szklarczyk D, Trachana K, Roth A, Huerta-Cepas J, Gabaldon T, Rattei T, Creevey C, Kuhn M et al. 2014. eggNOG v4.0: nested orthology inference across 3686 organisms. *Nucleic acids research* **42**(Database issue): D231-239.

Qin J, Li R, Raes J, Arumugam M, Burgdorf KS, Manichanh C, Nielsen T, Pons N, Levenez F, Yamada T et al. 2010. A human gut microbial gene catalogue established by metagenomic sequencing. *Nature* **464**(7285): 59-65.

Qin N, Yang F, Li A, Prifti E, Chen Y, Shao L, Guo J, Le Chatelier E, Yao J, Wu L et al. 2014. Alterations of the human gut microbiome in liver cirrhosis. *Nature* **513**(7516): 59-64.

Scher JU, Sczesnak A, Longman RS, Segata N, Ubeda C, Bielski C, Rostron T, Cerundolo V, Pamer EG, Abramson SB et al. 2013. Expansion of intestinal Prevotella copri correlates with enhanced susceptibility to arthritis. *Elife* **2**: e01202.

Sunagawa S, Coelho LP, Chaffron S, Kultima JR, Labadie K, Salazar G, Djahanschiri B, Zeller G, Mende DR, Alberti A et al. 2015. Ocean plankton. Structure and function of the global ocean microbiome. *Science* **348**(6237): 1261359.

Tringe SG, Rubin EM. 2005. Metagenomics: DNA sequencing of environmental samples. *Nat Rev Genet* **6**(11): 805-814.

Tringe SG, von Mering C, Kobayashi A, Salamov AA, Chen K, Chang HW, Podar M, Short JM, Mathur EJ, Detter JC et al. 2005. Comparative metagenomics of microbial communities. *Science* **308**(5721): 554-557.

Villar E, Farrant GK, Follows M, Garczarek L, Speich S, Audic S, Bittner L, Blanke B, Brum JR, Brunet C et al. 2015. Ocean plankton. Environmental characteristics of Agulhas rings affect interocean plankton transport. *Science* **348**(6237): 1261447.

Yang Y, Li B, Ju F, Zhang T. 2013. Exploring Variation of Antibiotic Resistance Genes in Activated Sludge over a Four-Year Period through a Metagenomic Approach. *Environ Sci Technol* **47**(18): 10197-10205.

Yok NG, Rosen GL. 2011. Combining gene prediction methods to improve metagenomic gene annotation. *BMC Bioinformatics* **12**: 20.

Zeller G, Tap J, Voigt AY, Sunagawa S, Kultima JR, Costea PI, Amiot A, Bohm J, Brunetti F, Habermann N et al. 2014. Potential of fecal microbiota for early-stage detection of colorectal cancer. *Mol Syst Biol* **10**: 766.

Zhu W, Lomsadze A, Borodovsky M. 2010. Ab initio gene identification in metagenomic sequences. *Nucleic acids research* **38**(12): e132.
